# Metabolite Profiling to Evaluate Metabolic Changes in Genetically Modified Protopanaxadiol-Enriched Rice

**DOI:** 10.3390/plants12040758

**Published:** 2023-02-08

**Authors:** Ji-Eun Sim, Sung-Dug Oh, Kiyoon Kang, Yu-Mi Shin, Doh-Won Yun, So-Hyeon Baek, Yong-Eui Choi, Sang-Un Park, Jae-Kwang Kim

**Affiliations:** 1 Division of Life Sciences and Convergence Research Center for Insect Vectors, Incheon National University, Incheon 22012, Republic of Korea; 2 National Institute of Agricultural Sciences, Rural Development Administration (RDA), Wanju-gun 55365, Republic of Korea; 3 Department of Agricultural Life Science, Sunchon National University, 255, Jeonnam 57922, Republic of Korea; 4 Department of Forest Resources, College of Forest and Environmental Sciences, Kangwon National University, Chuncheon 24341, Republic of Korea; 5 Department of Crop Science and Department of Smart Agriculture Systems, Chungnam National University, 99 Daehak-ro, Daejeon 34134, Republic of Korea

**Keywords:** metabolic profiling, multivariate analysis, transgenic rice, protopanaxadiol rice, protopanaxadiol, *PgDDS*, *CYP716A47*

## Abstract

Event DS rice producing protopanaxadiol (PPD) has been previously developed by inserting *Panax ginseng* dammarenediol-II synthase gene (*PgDDS*) and PPD synthase gene (*CYP716A47*). We performed a gas chromatography–mass spectrometry (GC–MS)-based metabolomics of the DS rice to identify metabolic alterations as the effects of genetic engineering by measuring the contents of 65 metabolites in seeds and 63 metabolites in leaves. Multivariate analysis and one-way analysis of variance between DS and non-genetically modified (GM) rice showed that DS rice accumulated fewer tocotrienols, tocopherols, and phytosterols than non-GM rice. These results may be due to competition for the same precursors because PPDs in DS rice are synthesized from the same precursors as those of phytosterols. In addition, multivariate analysis of metabolic data from rice leaves revealed that composition differed by growth stage rather than genetic modifications. Our results demonstrate the potential of metabolomics for identifying metabolic alterations in response to genetic modifications.

## 1. Introduction

Rice (*Oryza sativa* L.) is extensively cultivated worldwide and is consumed by more than half of the world’s population [1]. Rice crops can easily absorb the three major nutrients (carbohydrates, proteins, and fats), minerals and vitamins. Rice has been widely cultivated using genetic engineering to increase its nutrients and productivity. In particular, genetically modified (GM) rice has been extensively studied, focusing on developing functional rice with additional specific nutrients [2].

*Panax ginseng* is a well-known widely used medicinal plant. Dammarane-type triterpenoid saponins are the main pharmacologically active components of ginseng. Dammarane-type ginsenosides are classified into protopanaxadiol (PPD) and protopanaxatriol (PPT) based on their aglycone structures [3]. PPD is a ginsenoside intermediate existing in trace or undetectable amounts on ginseng root [4]. PPD exhibits various pharmacological activities with wide anti-cancer properties and is also involved in immune system regulation [5]. The artificial PPD collection from ginseng is challenging as it requires complex deglycosylation through enzymatic and chemical–physical treatments [4]. Therefore, to facilitate the absorption of medicinal components, we previously developed event DS rice that synthesizes PPD by overexpressing *Panax ginseng* dammarenediol-II synthase gene (*PgDDS*) and protopanaxadiol synthase gene (*CYP716A47*) driven by a rice endosperm-specific α-globulin promoter [6]. PgDDS and CYP716A47 are enzymes that activate the synthesis from squalene to PPD [7]. In a previous study, Han et al. (2019) demonstrated that PPD is well expressed in DS rice seeds. However, PPD was not detected in non-GM rice seeds. Ginseng is mainly used when the root is 4–6 years old, and its pharmacological components are obtained in the form of ginsenosides, not PPD. However, because the cultivation period of rice is approximately 6–7 months, pharmacological components in the form of PPD can be ingested rapidly [6,8].

A metabolomic approach can be used to evaluate the compositional changes in the developed GM rice. Metabolomics is a research field that investigates metabolic associations by identifying metabolite changes and characteristics [9]. Correlations between metabolites are the net result of enzymatic changes and cellular control of the transcriptional or biochemical events. Metabolomic approaches, including principal component analysis (PCA) and partial least squares discriminant analysis (PLS-DA), are widely used for separating samples with biological status, quality, or genetic differences [10]. Metabolomics has been steadily used to identify intended compositional changes in GM crops [9,11,12]. Thus, the metabolomics approaches have been developed to identify predicted or unpredicted changes in metabolic networks and have become an important tool in safety assessment of GM plants. Recently, the application of metabolomics methodology has been shown to be useful for the evaluation of unintended changes to chemical compositions in GM plants such as resveratrol-enriched rice, β-carotene-enhanced soybean, and β-carotene-enhanced rice [9,11,13]. However, to the best of our knowledge, metabolomics studies of rice synthesizing PPD have not yet been conducted. Therefore, the metabolic changes resulting from the genetic modification of DS rice must be evaluated. In this study, metabolomics-based identification of the metabolic alterations in DS rice was performed.

## 2. Results and Discussion

### 2.1. Multivariate Analysis of Rice Seed

Metabolic profiling of control Dongjin rice (DJ) and GM rice (DS1 and DS8) seeds was performed by analyzing the hydrophilic and lipophilic compounds. Sixty-five metabolites were identified, including phytosterols, tocopherols, policosanols, amino acids, sugars, sugar alcohols, and organic acids in rice seeds (Tables S1 and S2). The metabolite information was submitted to MetaboLights public repository (database no. MTBLS6937). Multivariate analysis, which is used to distinguish patterns in various datasets of results [14], was performed to compare metabolite differences.

**Figure 1:**
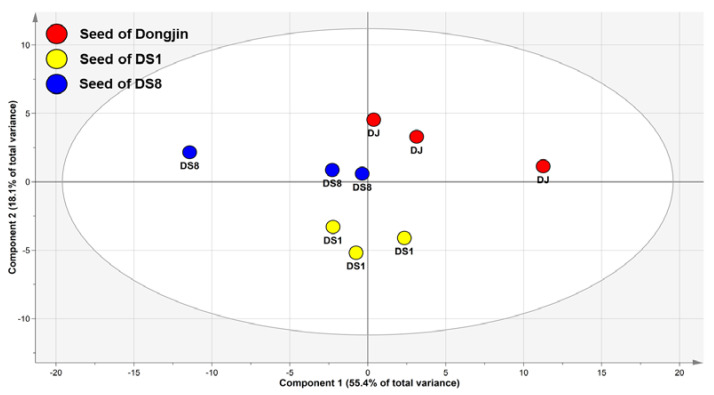
PCA score plot on rice seed. Abbreviations: DJ, *Oryza sativa* L. cv. Dongjin; DS1, line 1 of transformed rice from DJ with *PgDDS* and *CYP716A47* genes; DS8, line 8 of transformed rice from DJ with *PgDDS* and *CYP716A47* genes.

**Figure 2:**
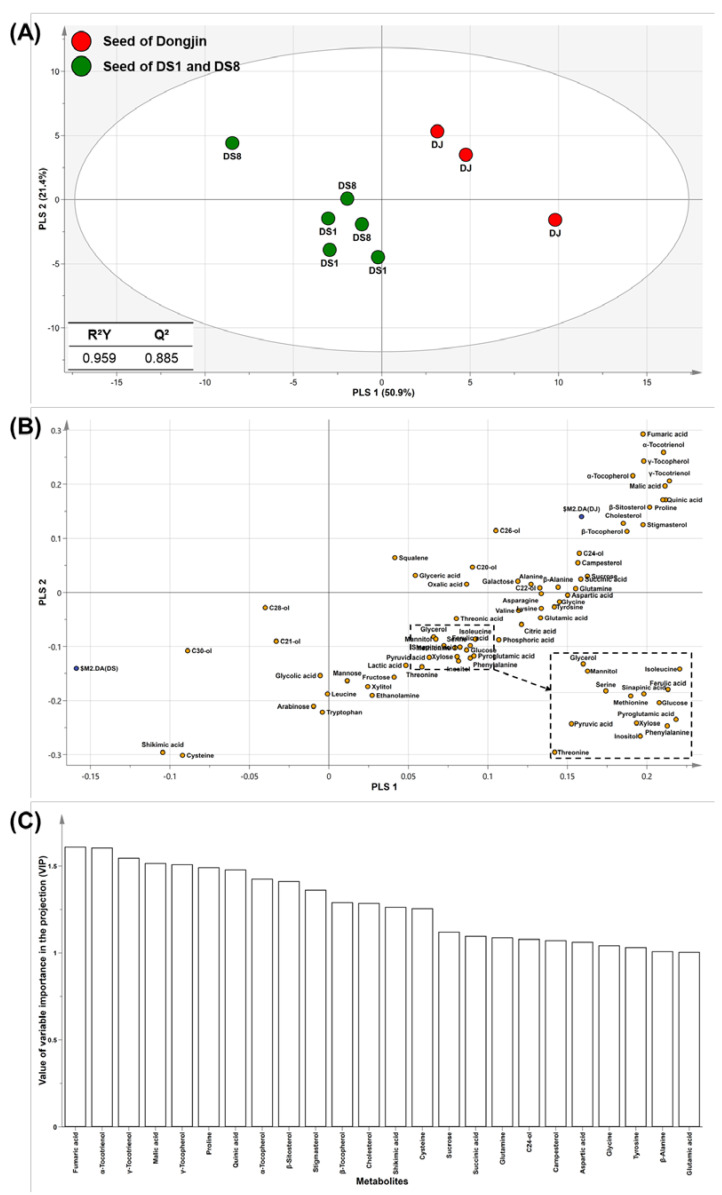
Score (**A**), loading (**B**) plots and variable importance in the projection (VIP) (**C**) of the PLS-DA models on rice seed. Abbreviations: DJ, *Oryza sativa* L. cv. Dongjin; DS1, line 1 of transformed rice from DJ with *PgDDS* and *CYP716A47* genes; DS8, line 8 of transformed rice from DJ with *PgDDS* and *CYP716A47* genes; C20-ol, eicosanol; C21-ol, heneicosanol; C22-ol, docosanol; C24-ol, tetracosanol; C26-ol, hexacosanol; C28-ol, octacosanol; C30-ol, triacontanol.

PCA can be used to assess differences in samples based on their metabolite levels. In this PCA model, two high-ranking principal components (PC) explained for 73.5% of the total variance within the dataset (PC1, 55.4%; PC2, 18.1%) (Figure 1). Although clustered by groups (DJ, DS1, and DS8), the DJ and DS rice seeds were not separated in PC1 of the score plot. Therefore, a PLS-DA model was generated to identify metabolic differences (Figure 2A).

PLS-DA rotates the PCA projection to obtain the maximum separation of variables by class [1]. In this study, DJ, DS1, and DS8 rice were used as classes for model validation. R^2^ and Q^2^ are validation parameters that represent the model quality. R^2^ specifies the ratio of variation in data provided by the model, and Q^2^ specifies the ratio of variation in data predicted by the model. An R^2^ value closer to 1 indicates a good fit for the prediction model, and Q^2^ > 0.5 indicates a good predictive ability [1]. Our PLS-DA model showed an R^2^Y of 0.959 and Q^2^ of 0.885. DJ rice and the two DS rice were clustered separately by PLS1. These results reflect intentional changes caused by the genetic modification of rice seeds. To identify the metabolites contributing to separation, a loading plot was generated (Figure 2B), which showed that the levels of most metabolites (such as sucrose, sugar alcohols, amino acids, phytosterols, and tocopherols) were positive for PLS1. Metabolites with positive values in PLS1 were present at higher levels in DJ rice seeds. The value of variable importance in the projection (VIP) was used to confirm the contribution of the PLS-DA model metabolites (Figure 2C). VIP values > 1 indicated a significant contribution to the model. Metabolites such as fumaric acids, proline, tocotrienols, tocopherols and phytosterols were highly ranked in the VIP plot. Highly ranked metabolites of VIP significantly contributed to the separation between DJ and DS rice.

### 2.2. Tocopherol and Phytosterol Content in Rice Seed

**Figure 3:**
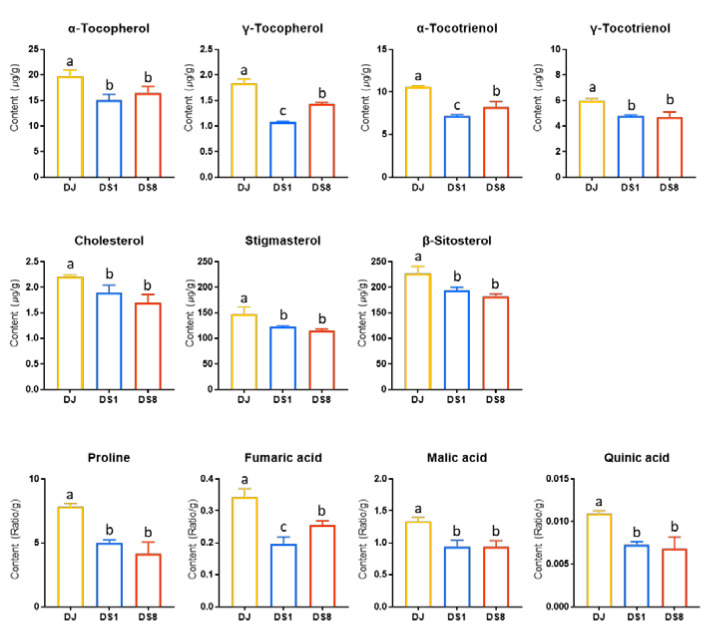
Contents of DJ, DS1, and DS8 rice metabolites with significant differences in ANOVA. Bar graphs with different letters (a, b, c) refer to the significant differences between the samples, as obtained by one-way ANOVA. Error bars represent standard deviations. Abbreviations: DJ, *Oryza sativa* L. cv. Dongjin; DS1, line 1 of transformed rice from DJ with *PgDDS* and *CYP716A47* genes; DS8, line 8 of transformed rice from DJ with *PgDDS* and *CYP716A47* genes.

**Figure 4:**
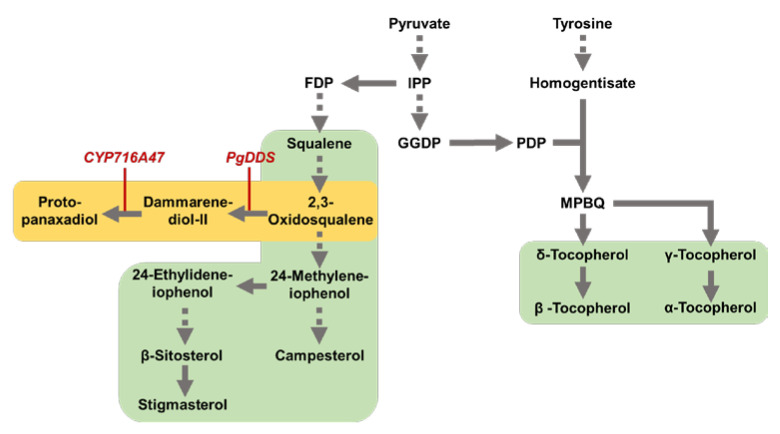
Biosynthetic pathway for protopanaxadiol, phytosterols, and tocopherols. Abbreviations: IPP, isopentenyl diphosphate; FDP, farnesyl diphosphate; GGDP, geranylgeranyl diphosphate; PDP, phytyl diphosphate; MPBQ, 2-methyl-6-phytyl-1,4-benzoquinol.

To confirm the significant difference (*p* < 0.05) in metabolites between DJ and DS rice, one-way analysis of variance (ANOVA) was performed for metabolites with VIP values > 1.0 in Figure 2C. The false discovery rate (FDR) method was used to control for false positive outcomes across the analytes (Table S3). Significant differences (*p* < 0.05) in the metabolites of DJ, DS1, and DS8 rice were identified using bar graphs. Eleven metabolites were significantly different between DJ and DS rice (Figure 3). Of these, seven were lipid metabolites (including α-tocopherol, γ-tocopherol, α-tocotrienol, γ-tocotrienol, cholesterol, stigmasterol, and β-sitosterol). DJ rice produced higher levels of tocopherols, tocotrienols, and phytosterols than DS rice, indicating that fewer tocopherols, tocotrienols, and phytosterols were synthesized in DS rice that synthesizes PPD. In DS rice, *PgDDS* is expressed to synthesize dammarenediol-II from 2,3-oxidosqualene, and then *CYP716A47* is expressed to synthesize PPD (Figure 4). PPD synthesis is a part of the mevalonate (MVA) pathway [6]. Similar to PPD, phytosterols are synthesized by the MVA pathway [15]. Therefore, PPD synthesis is correlated with phytosterol suppression. In a previous study, transgenic tobacco synthesizing PPD through the recombination of *PgDDS* and *CYP716A47* decreased the phytosterol (stigmasterol, β-sitosterol, and campesterol) content. Because PPD and phytosterols are synthesized from the same precursor, 2,3-oxidosqualene, the lower phytosterol contents in transgenic rice may be due to the competition for precursors [7]. Our results were consistent with those of previous study [7]. In our study, phytosterol and tocopherol contents were less accumulated in DS rice, presumably due to the high precursor consumption during PPD synthesis. Furthermore, these results suggest that the contents of tocotrienols, tocopherols, and phytosterols in DJ and DS rice differ owing to the influence of genetic modification.

### 2.3. Multivariate Analysis of Rice Leaves

Rice leaves synthesize and store energy from photosynthesis and deliver it to storage organs, such as seeds. Therefore, owing to the close correlation between leaves and seeds, metabolite profiling of rice leaves was also performed by analyzing the hydrophilic and lipophilic compounds at different growth stages (8, 12, and 16 weeks). Sixty-three metabolites, including phytosterols, tocopherols, policosanols, amino acids, sugars, sugar alcohols, and organic acids, were identified in rice leaves (Tables S4 and S5). Multivariate analysis was conducted to establish differences in metabolite composition by genotype and growth stage (leaves of DJ and DS rice at 8, 12, and 16 weeks).

**Figure 5:**
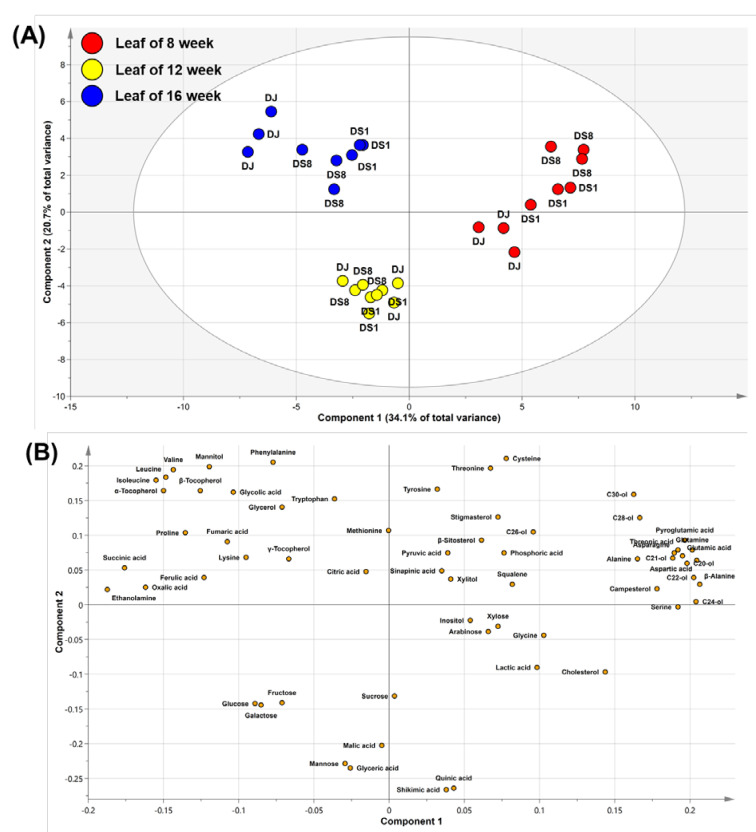
Score (**A**) and loading (**B**) plots of the PCA on rice leaves. Abbreviations: DJ, *Oryza sativa* L. cv. Dongjin; DS1, line 1 of transformed rice from DJ with *PgDDS* and *CYP716A47* genes; DS8, line 8 of transformed rice from DJ with *PgDDS* and *CYP716A47* genes; C20-ol, eicosanol; C21-ol, heneicosanol; C22-ol, docosanol; C24-ol, tetracosanol; C26-ol, hexacosanol; C28-ol, octacosanol; C30-ol, triacontanol.

In the PCA model, two PCs represented 54.8% of the total variance (PC1, 34.1%; PC2, 20.7%) (Figure 5A). The PCA results of the leaves were separated by growth stage rather than by genotype. A loading plot was created to identify the contribution of growth stages to metabolite separation (Figure 5B). Rice leaves at 8 weeks showed higher levels of policosanols and phytosterols than those at 12 and 16 weeks. At 12 weeks, rice leaves had high levels of monosaccharides, sucrose, malic acid, and quinic acid. At 16 weeks, rice leaves showed higher levels of proline, TCA cycle intermediates (succinic acid and fumaric acid), and tocopherols than at 8 and 12 weeks.

In this study, the rice was cultivated through the rice transplantation method. Unlike other plants, the transplantation method has mainly been used for rice cultivation as it prevents uneven germination and cold-heat damage in the initial stages of growth and is easy to manage weeds [16]. Rice is usually transplanted between 4 and 6 weeks after sowing them in seedling beds [17]. However, transplantation results in transplantation shock due to root damage and rapid changes in growth conditions. Changes in water content due to root damage can temporarily disrupt metabolic processes in transplanted seedlings and produce metabolites involved in stress response [18]. The increase in phytosterol and policosanol contents in 8-week-old leaves is presumed to be due to the transplantation stress response. Phytosterols are involved in biotic and abiotic stress responses in plants; their accumulation in plants suggests their role in providing tolerance to stress [19]. In addition, stress due to transplantation shock can affect plastid development by downregulating its activity as a plant defense response [18,20]. Our data revealed that tocopherol accumulation was minimal in 8-week-old leaves, suggesting that the non-mevalonate (MEP) pathway inside the plastid is downregulated [18,21].

After rice transplantation, the growth and development of seedlings gradually become more active after the restoration period [16]. Plants produce and store large amounts of energy through photosynthesis. During the growing stage, young leaves fix a large amount of carbon through photosynthesis, which is then used as an energy source in the form of monosaccharides for plant growth [22]. Meanwhile, organic acids such as oxalic acid, succinic acid, and citric acid are either synthesized into sucrose through gluconeogenesis or oxidized through the TCA cycle [23]. In this study, rice leaves during the restoration period produced large amounts of mono-saccharides and sucrose at 12 weeks. The 16-week-old rice, which is in the heading stage, actively synthesized energy through photosynthesis to promote grain growth [24]. Plastids were activated for photosynthesis, and a large amount of tocopherol was accumulated by the MEP pathway in 16-week-old leaves. Unlike other plants, transplanted rice leaves have different amounts of metabolites depending on the growth stage. As a result, rice leaves are presumed to be affected by the external environment or growth stage rather than by genetic modification.

## 3. Materials and Methods

### 3.1. Rice Sample Preparation

DS rice was prepared by inserting the dammarenediol-II synthase gene (*PgDDS*, GenBank: GU183405.1) and dammarenediol-II 12-hydroxylase gene (*CYP716A47*, GenBank: JN604536.1) into *Oryza sativa* L. cv. DJ rice [6]. The *PgDDS* and *CYP716A47* genes were derived from the *Panax ginseng* roots. Rice was sown in seedling beds in May, and transplanted into rice fields in June using the rice transplantation method. Rice was cultivated in an isolated living modified organism (LMO) experimental area (facility registration number: RDA-AB-2013-041) at the National Institute of Agricultural Sciences located in Jeonju (latitude: 35°49′51″ N; longitude: 127°03′55″ E), Republic of Korea. Rice leaves were collected at 8, 12, and 16 weeks, and seeds were harvested at 27 weeks. DS rice expresses PPD only in seeds [6]. Two lines (DS1 and DS8) that adequately express PPD were selected based on their gene expression levels. Samples were converted into powder using a blender (HR2860; Philips, Amsterdam, Netherlands) and stored at −20 °C. PPD in DS seeds were analyzed as previously described [6]. Milled powders (100 mg) from rice grains were soaked in 100% methanol and sonicated for 30 min at 40 °C. The supernatant after centrifugation was collected before injection. Analysis was performed with a Shimadzu LC system (Kyoto, Japan) equipped with a binary pump (LC-20AD), a degasser (DGU-20A), an autosampler (SIL-20A), a column oven (CTO-20AC), a PDA detector (SPD-M20A) on a YMC-Pack Pro C18 RS column (150 × 2.0 mm. D, S-5 µm, 8 nm, YMC Co., Ltd., Kyoto, Japan) at 40 °C. The liquid chromatograph–mass spectrometry ion trap/time-of-fight (LCMS–IT-TOF) (Shimadzu, Kyoto, Japan) was equipped with an atmospheric pressure chemical ionization (APCI) source in the positive and/or negative ion modes. Authentic PPD was directly subjected to the same conditions (Figure S1). The mean PPD concentrations in DS1 rice seeds were 13.00 µg/g dry weight and in DS8 rice seeds were 14.96 µg/g dry weight.

### 3.2. Extraction and Analysis of Hydrophilic Metabolites

Hydrophilic compounds (free amino acids, sugars, sugar alcohols, and organic acids) were analyzed as previously described (Table S6) [9]. A total of 10 mg of ground rice samples were added in a 2 mL tube with 1 mL of methanol:water:chloroform (2.5:1:1, *v/v/v*) solution. Next, 0.06 mL of ribitol (0.2 mg/mL in methanol) was added as an internal standard (IS). After vortexing, the mixtures were cultured in a thermomixer (5355 model, Eppendorf AG, Hamburg, Germany) with shaking at 1200 rpm and 37 °C for 30 min. The mixtures were centrifuged at 16,000× *g* at 4 °C for 5 min. The 0.8 mL of supernatant was transferred to a new 2 mL tube, and then 0.4 mL of deionized water was added. After vortexing, the samples were centrifuged at 16,000× *g* and 4 °C for 5 min, and then 0.9 mL of supernatant was transferred to a new 2 mL tube, incubated with a centrifugal concentrator (CC-105, TOMY, Tokyo, Japan) for 4 h and freeze-dried at −80 °C for 16 h. For methoxime derivatization, 0.08 mL of methoxyamine hydrochloride (MOX, 20 mg/mL) in pyridine was added in samples and cultured with shaking at 1200 rpm and 30 °C for 90 min. Next, 0.08 mL of N-methyl-N-(trimethylsilyl) trifluoroacetamide (MSTFA) was treated, and the samples were incubated with shaking at 1200 rpm and 37 °C for 30 min. The sample was transferred to the insert in a gas chromatography (GC) auto sampler glass vial. The hydrophilic metabolites were analyzed with GC-TOF-MS using an Agilent 7890 B GC (Agilent, Santa Clara, CA, USA) with a Pegasus GC-TOF-MS Benchtop (LECO, St. Joseph, MI, USA). The Rtx-5MS column (30 m × 0.25 mm, 0.25-μm i.d. film thickness; Restek, Bellefonte, PA, USA) was equipped in the GC, and the helium gas flow rate was set at 1 mL/min. Then, 0.001 mL of the sample extract was injected in 1:25 ratio split mode. The inlet temperature was set at 230 °C. The oven temperature was set initially at 80 °C for 2 min, followed by ramping to 320 °C (15 °C/min) and holding for 10 min. The ion source and transfer line temperatures were set to 250 °C and 280 °C, respectively. The spectral data were scanned at 85–600 *m/z*. We confirmed hydrophilic compounds by standards (MSI level 1) in targeted metabolite profiling [25]. ChromaTOF software (LECO, St. Joseph, MO, USA) was used to identify the hydrophilic compounds in rice. The Chroma TOF software package was used to extract raw peaks, filter and calibrate data baselines, align peaks, perform deconvolution analysis, identify peaks, and integrate peak areas. For quantification, the ratio of the relative peak area to that of the IS was determined based on the selected ions.

### 3.3. Extraction and Analysis of Lipophilic Metabolites

Lipophilic compounds (policosanols, tocotrienols, tocopherols, and phytosterols) were detected using a previously described method (Table S7) [1]. Ground samples of 100 mg rice seeds and 20 mg rice leaves were extracted containing 3 mL of 0.1% ascorbic acid in ethanol (*w/v*) in a 15 mL tube. Next, 0.05 mL of 5α-cholestane (10 µg/mL) was added as the IS. After vortexing, the mixture was cultured in 85 °C water basket for 5 min. A total of 0.12 mL of potassium hydroxide (80%, *w/v*) was added for saponification and vortexed. The mixture was then cultured in 85 °C water basket for 10 min. The solution was immediately placed in an ice basket for 5 min, and 1.5 mL of deionized water and hexane was added. Subsequently, the solution was mixed for 20 s before centrifugation at 1200× *g* and 4 °C for 5 min. The supernatant of the solution was transferred to a new 15 mL tube. Hexane (1.5 mL) was added for re-extraction. The hexane fraction was collected in 15 mL tubes. The solution was evaporated under nitrogen gas, and concentrated using the centrifugal concentrator. For derivatization, 0.03 mL of MSTFA and pyridine were added, respectively, and the tube was incubated with shaking at 1200 rpm and 60 °C for 30 min. The lipophilic metabolites were identified using a GC-MS QP2010 Ultra system (GC-qMS) installed with the Rtx-5MS column equipped with an autosampler (AOC-20i, Shimadzu, Kyoto, Japan). The 0.001 mL of sample was injected in 1:10 split mode at an injection inlet temperature of 290 °C. The carrier gas was helium, and the gas flow rate was 1.00 mL/min. The oven temperature was maintained at 150 °C for 2 min, subsequently increased up to 320 °C (15 °C/min) and holding for 10 min. The MS ion source temperature was 230 °C, and the interface temperature was 280 °C. The mass spectra range was 85–600 *m/z*. Peak analysis was executed in the selected ion monitoring mode. Chromatography and mass spectra were obtained using Lab Solutions GC-MS solution software (4.20 version, Shimadzu, Kyoto, Japan). Qualitative and quantitative analyses were performed as described by our group [26]. Quantification was performed by means of tree-point calibration curves, for which the concentrations of authentic standards ranged from 0.25 to 5.00 μg.

### 3.4. Statistical Analysis

Three biological replicates of rice samples were used per group. PCA and PLS-DA were used to analyze metabolites with the SIMCA software (version 14.1, Umetrics, Umeå, Sweden). Data were normalized using unit variance scaling before multivariate data analysis. A score plot of PCA and PLS-DA was used to visualize the sample grouping, and loading plots supported the classification of groups in score plots. To estimate the differences between rice contents, ANOVA combined with Duncan’s multiple range test was performed using the statistical analysis program SAS enterprise guide 7.1 (SAS Institute Inc., Cary, NC, USA). Additionally, the FDR adjustment of raw *p*-values was conducted by using ‘MetaboAnalyst5.0’ (https://www.metaboanalyst.ca (accessed on 1 January 2023)). Differences were considered significant if the FDR-adjusted *p*-value was less than 0.05.

## 4. Conclusions

In this study, we performed metabolic profiling of DJ and DS rice. We obtained results for metabolic profiling of saponin biosynthesis in rice for the first time. PPD was detected in DS rice overexpressing *PgDDS* and *CYP716A47* genes but not in DJ rice. Multivariate analysis of metabolite profiles segregated rice seeds based on genetic differences. Tocopherols, tocotrienols, and phytosterols contributed to this separation. ANOVA test revealed significant differences between DJ and DS rice, with DS rice having lower tocopherol, tocotrienol, and phytosterol contents than DJ rice. Because PPD and phytosterols are synthesized from squalene, the lower phytosterol content in DS rice may be due to competition for the precursor. In addition, rice leaves were separated according to their growth stages rather than by genetic modification. Because PPD is synthesized specifically in seeds, rice leaves are thought to be affected by natural variability rather than by genetic modification. Overall, these results demonstrate that metabolic profiling can be used to assess the effects of genetic modification. Furthermore, metabolic profiling can reflect the natural variability of metabolites associated with the environment. Therefore, targeted metabolite profiling is suggested as an appropriate analytical tool for the intended and unintended metabolic changes in GM rice. Additionally, it can provide valuable information for rice cultivar development.

## Data Availability

The data presented in this study are contained within the article or Supplementary Materials.

## Supplementary Materials

The following supporting information can be downloaded at: https://www.mdpi.com/article/10.3390/plants12040758/s1. Table S1. Composition of hydrophilic compounds in rice seeds; Table S2. Composition of lipophilic compounds in rice seeds; Table S3. FDR-adjusted p-values and non-adjusted p-values of compositions in DJ and DS rice seeds; Table S4. Composition of hydrophilic compounds in rice leaves; Table S5. Composition of lipophilic compounds in rice leaves; Table S6. Relative retention times (RRT) and mass spectrometry data of hydrophilic compounds in rice seeds and leaves; Table S7. Relative retention times (RRT) and mass spectrometry data of lipophilic compounds in rice seeds and leaves. Figure S1. LCMS-IT-TOF analyses of grain extracts from transgenic rice co-overexpressing PgDDS and CYP716A47. (A) Selected ion mode (SIM) chromatogram of the methanol extracts of the non-transgenic rice grains. (B) SIM chromatogram of PPD signal at 25.5 min in the extracts of transgenic rice grains. (C) SIM chromatogram of the authentic standards (PPD). (D) Positive ionization of the standard PPD peak. (E) Positive ionization of PPD peak in a transgenic rice extract.
